# Supplementary material for: Ferroptosis and Apoptosis Are Involved in the Formation of L-Selenomethionine-Induced Ocular Defects in Zebrafish Embryos
Source: Int J Mol Sci. 2022 Apr 26;23(9):4783. doi: 10.3390/ijms23094783 (PMC9100823; doi:10.3390/ijms23094783)
Supplement: Supplementary file 1 [file ijms-23-04783-s001.zip › Supplementary figures.pdf]

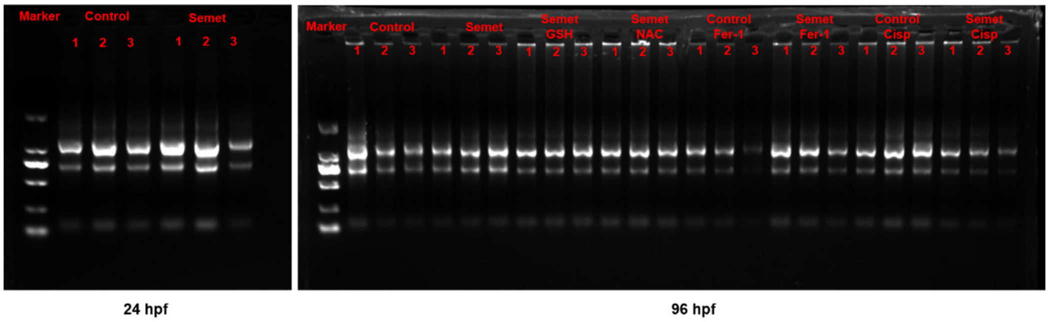

Figure S1. Agarose gel electrophoresis of RNA used in this study.

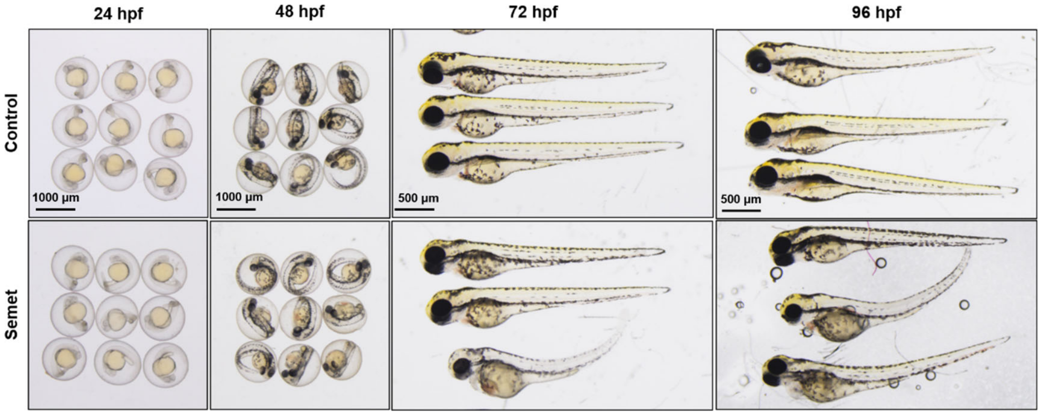

Figure S2. Phenotypes of representative selenium-stressed embryos at 24, 48, 72 and 96 hpf.

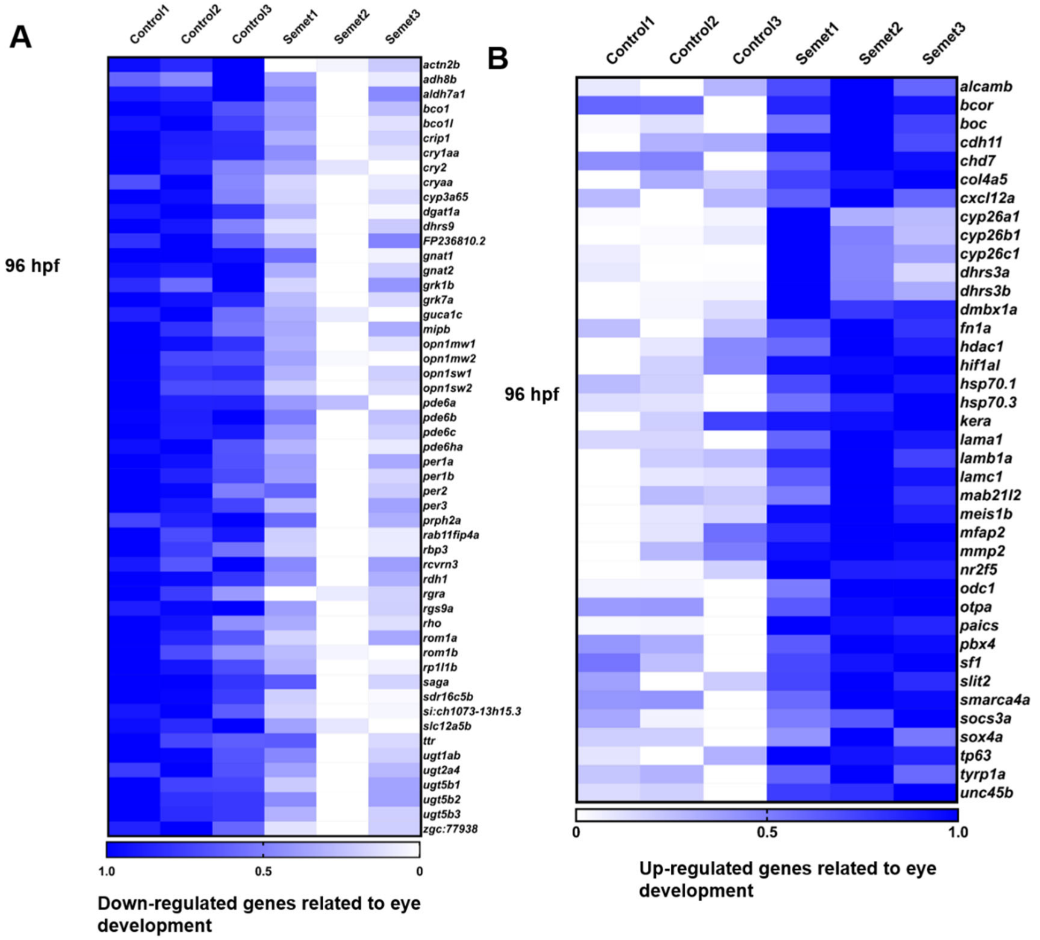

Figure S3. Heatmaps of genes related to eye development (A, down-regulation; B, upregulation) based on RNA-seq data at 96 hpf.

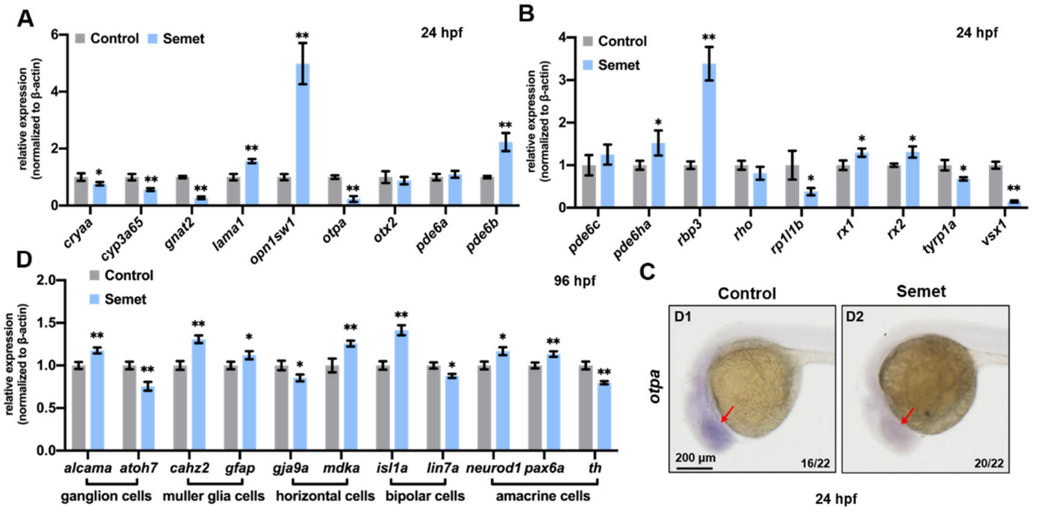

Figure S4. Expression of ocular marker genes in selenium-treated embryos. (A, B) The expression of ocular marker genes in selenium-treated embryos at 24 hpf detected by qRT-PCR. (C) The expression of

ganglion, muller glia, horizontal, bipolar and amacrine cell marker genes at 96 hpf. (D) WISH data of *otpa* (D3-D4) in control and selenium-treated embryos at 24 hpf.

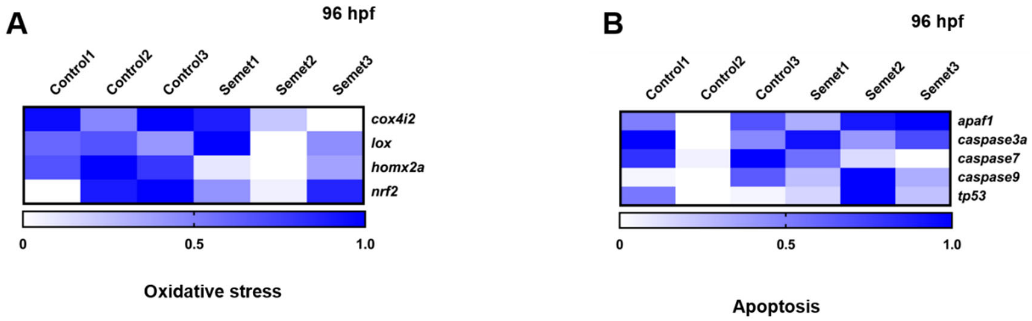

Figure S5. Heatmaps of genes related to oxidative stress (A) and apoptosis (B) based on RNA-seq data at 96 hpf.

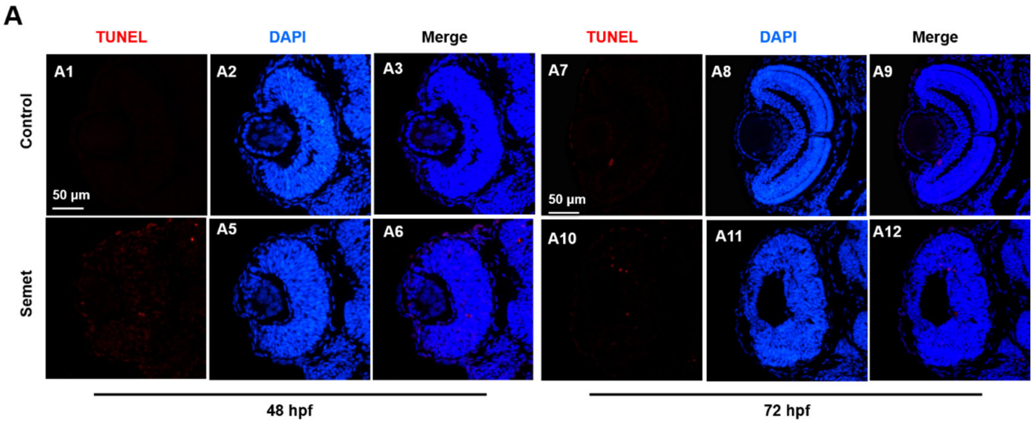

Figure S6. Cell apoptosis detected by TUNEL assay in control and selenium-treated embryos at 48 hpf and 72 hpf.

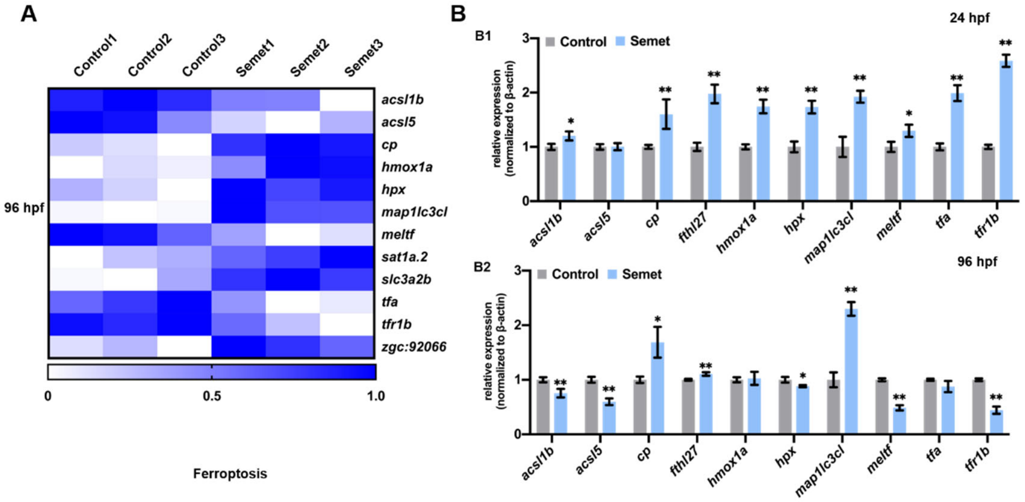

Figure S7. Expression of ferroptosis marker genes in selenium-treated embryos. (**A**) DEGs of ferroptosis detected by RNA-seq in selenium-treated embryos at 96 hpf. (**B**) The expression of ferroptosis marker genes in selenium-treated embryos at 24 hpf (**B1**) and 96 hpf (**B2**) detected by qRT-PCR.
